# Phenolic Profile, Antioxidant Activity, and Anti-obesogenic Bioactivity of Mao Luang Fruits (*Antidesma bunius* L.)

**DOI:** 10.3390/molecules24224109

**Published:** 2019-11-14

**Authors:** Ornnicha Krongyut, Khaetthareeya Sutthanut

**Affiliations:** 1 Department of Pharmaceutical Chemistry, Faculty of Pharmaceutical Sciences, Khon Kaen University, Khon Kaen 40002, Thailand; belle_bigbang@hotmail.com; 2 Human High Performance & Health Promotion Research Institute: HHP&HP Research Institute, Khon Kaen University, Khon Kaen 40002, Thailand

**Keywords:** *Antidesma bunius* L., anti-obesity, α-amylase, pancreatic lipase, adipocyte, anti-adipogenesis

## Abstract

To investigate the anti-obesity potential of *Antidesma bunius* L. (MM), a Thai local fruit which is named “Mao Luang,” we have focused on the effects on pancreatic α-amylase and lipase enzyme activity and on adipocyte life cycle using the 3T3-L1 cell line as a model. In addition, the phytochemical composition and anti-oxidation potential were also analyzed using HPLC-PDA UV and colorimetric methods. The ethanolic extract of MM fruits prepared by a maceration method was used in the experiments. MM extract, yield 12.08% *w*/*w*, is composed primarily of phenolics and anthocyanins as the major phytochemicals, among which, gallic acid, catechin, anthocyanin-3-glucoside, and protocatechuic acid were initially identified. In addition, susceptibly inhibitory effects on oxidation in a DPPH assay; on lipase enzyme activity rather than amylase enzyme; and on adipocyte adipogenesis of MM were demonstrated. Interestingly, a concentration-dependent bi-modular manner of activity on adipocyte adipogenesis was discovered, whereby a significant anti-adipogenic effect was demonstrated at high concentration, whilst low concentrations of MM showed adipogenic induction. Lipolytic induction was manifested. Conclusively, the ethanolic MM extract was discovered to be a potential anti-obesity agent contributed by inhibitory effects on lipase enzyme and anti-differentiation and -adipogenesis in adipocytes which significantly correlated to the total phenolics content, as well as anti-oxidation as the mechanism of action. Nevertheless, to achieve effective application, further investigation in *in vivo* models should be considered.

## 1. Introduction

Obesity is a global concerning chronic disease due to its high incidence and prevalence [1] as well as being a risk factor in developing of other fatal chronic diseases, for instance, diabetes, high blood pressure and cardiovascular diseases [2]. The etiology of obesity involves multiple factors such as behavior, genetics, some medications, and diseases that finally lead to an imbalanced metabolism-induced excessive adipogenesis and lipid accumulation in tissues and organs. This begins at gastrointestinal food digestion and absorption involving amylase and lipase which are known as the two key enzymes responsible in carbohydrate and lipid digestion to produce glucose and glycerol and fatty acids, respectively [3,4]. These small molecules regularly undergo catabolism to produce energy for physiological activity and homeostasis, yet paradoxically, the overwhelming presence thereof in unbalanced conditions will lead to fat accumulation through adipogenesis, comprising pre-adipocyte differentiation and lipogenesis and lipid accumulation of mature adipocytes, which finally could result of overweight and obesity development [4]. Thus, inhibition of amylase and lipase enzymes in food digestion and on adipogenesis and induction of lipolysis in adipocytes have become targets of interest for obesity prevention and treatment [5]. Acarbose and orlistat are amylase and lipase inhibitors currently used in obesity therapy. In addition, other anti-obesity drugs related suppression of appetite and central nervous system must be used with caution due to their of serious adverse effects. Therefore, anti-obesity agents from natural sources have been identified as alternatives of interest with advantageous properties for applications with safe, multiple health promotion functions and less limited accessibility for all.

*Antidesma bunius* L. (family Phyllanthaceae), known as “Mao Luang” in Thai (MM), is a local fruit widely cultivated in Northeast Thailand. It is a shrub species with wide-spreading branches forming a dense crown evergreen. MM ripe fresh fruits are traditionally used for gastric intestinal and metabolic problems, e.g., diabetes, dysentery, indigestion and constipation, and for their cytotoxic, anti-diabetic, antioxidant, antiradical, thrombolytic activity, antiplatelet, anticoagulant, anti-dysenteric, antimicrobial, antihypertensive, anticancer and sudorific activity [6,7,8]. Recently, its nutrition, vitamins and minerals, phenolics, flavonoids, and anthocyanins components [8] and several bioactivities and functions, including anti-inflammation, prevention of free-radical-induced cell apoptosis, inhibition of α-glucosidase enzyme, and anti-diabetic activity, through the enhancement of hepatic glycogen storage and regeneration of the islet of Langerhans were reported [6,9,10]. However, evidence on the anti-obesity potential of MM is quite limited. Therefore, this study aimed to investigate the anti-obesity potential of MM ripe fruit, focusing on inhibition of pancreatic α-amylase and lipase enzyme function, anti-adipogenesis and lipolysis induction in 3T3-L1 adipocytes. The obtained data will provide supporting information in product development and applications of MM for health promoting and obesity prevention and treatment.

## 2. Results

### 2.1. The Different Phytochemical Contents and Anti-oxidation Properties Among the Samples

The extract of MM ripe fruits, obtained in 12.08% *w*/*w* yield (fresh weight), contained phenolics (11.6 ± 1.1 mg GA eq/g extract), flavonoids (0.30 ± 0.00 QE eq/g extract) and anthocyanins (3.76 ± 0.19 mg C3G eq/g extract). It exhibited anti-oxidation activity in both the DPPH assay (21.8 ± 3.5 mg Vit C eq/g extract) and TBAR assay (3.55 ± 0.42 mg BHT eq/g extract), however, these were found with different degrees. This was exhibited by the corresponding IC_50_ values which were 652 ± 5 µg/mL in the DPPH assay, but more than 1000 µg/mL in the TBARS assay (Table 1).

Moreover, based on the identical retention times and optical spectral data to the standard compounds, the chemical constituents of MM extract included gallic acid, catechin, cyanidin-3-glucoside and protocatechuic acid, as demonstrated by HPLC analysis. Among them, cyanidin-3-glucoside (13.4 ± 0.7 mg/g extract) and catechin (2.26 ± 0.61 mg/g extract) were detected as two predominant components of MM extract (Table 2). In addition, the optical spectra of other peaks have suggested their classes of phytochemicals including derivatives of phenolics (λ_max_ at 260–280 nm), flavonoids (λ_max_ at ~254 and ~350 nm), and antocyanins (λ_max_ at ~280 and ~520 nm) [11].

### 2.2. Inhibitory Activity Against α-Amylase and Lipase Enzymes

Different amylase and lipase enzyme inhibition activities were observed. The inhibitory effect on lipase enzyme of MM extract, rather than amylase enzyme, was demonstrated by giving IC_50_ 90.8 ± 4.1 µg/mL and equivalent weight of 14.3 ± 4.1 µg orlistat equiv./mg extract when compared to that obtained from amylase inhibition test. MM couldn’t achieve 50% amylase enzyme inhibition (only 44.2 ± 0.9%) at concentration 1000 µg/mL, giving an estimated acarbose equivalent weight as low as 0.45 ± 0.05 µg/mg extract. Compared to the reference drugs, orlistat and acarbose, the inferior potency of MM extract was displayed, being about ~70-times lower than orlistat. This means that to achieve an effect equivalent to orlistat, approximately a 70-fold higher concentration of MM extract is required (Table 3). This information implies the estimated proper dose of MM extract which should be accounted for in applications and product development.

### 2.3. Anti-obesity Potential via Interruption on Adipocyte Life Cycle

#### 2.3.1. Effect on Pre-adipocyte Viability by MTT Assay

Very mild to none cytotoxic effect on pre-adipocyte of MM extract was demonstrated at all tested concentrations, showing high viability of cells (with a range of 86.3 ± 2.5–106 ± 2%). Nevertheless a significant reduction of viability was detected at a concentration of 2000 µg/mL of MM extract when compared to the control, which had a % cell viability higher than 80. Therefore, a concentration range of 0–1000 µg/mL of MM extract was chosen for further experiments. 

#### 2.3.2. Anti-differentiation as Showing Reduction of Oil Red O Staining

**Figure 1:**
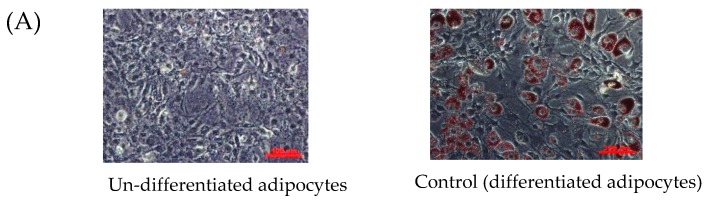

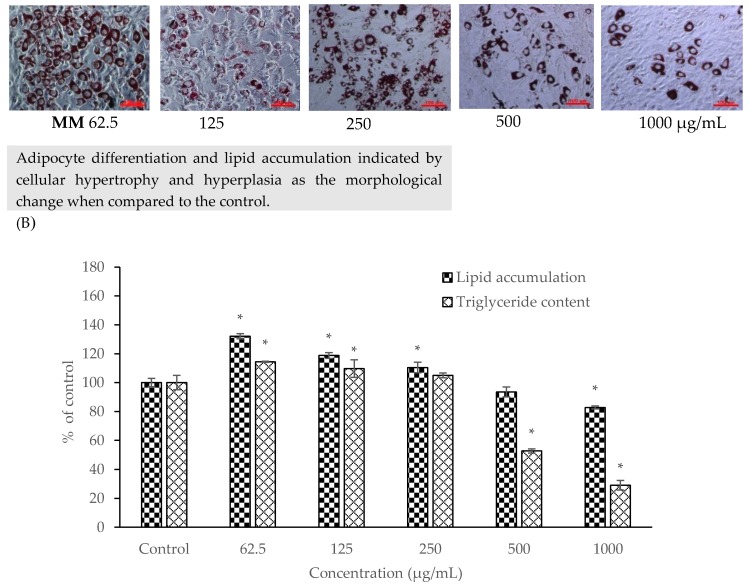
Bimodal effects on adipogenesis in a manner of concentration dependence of *Antidesma bunius* L. (MM) extract was displayed. Induction on pre-adipocyte differentiation and lipid accumulation at low concentrations (62.5–250 µg/mL) expressed by increasing of Oil Red O staining intensity which visualized the adipocyte hypertrophy and hyperplasia under inverted microscope (magnification 20×), in contrast, inhibitory effect at high concentration (500–1000 µg/mL) (**A**). In similar manner, the bimodal effects were demonstrated by total intracellular lipid accumulation and triglyceride content (**B**) with statistical significance (*) at *p*-value < 0.05 when compared to control (differentiated adipocytes).

Differentiation is a process of pre-adipocyte transformation into mature adipocytes which can be indicated by morphological changes-lipid-droplet-filled and rounded-shape cell-resulting from intracellular total lipid production and accumulation in the form of triacylglycerol (triglycerides). Based on the obtained results, a concentration-dependent bimodal effect of MM extract was revealed. When compared to the control, a slight induction of differentiation and lipid accumulation was detected at low concentration of MM extract with statistical significance at 62.5 and 125 µg/mL, showing of hypertrophic adipocytes as the morphological change indicated by incretion and expansion of Oil Red O stain (Figure 1A), whilst, inhibition on differentiation was found at high concentrations (500 and 1000 µg/mL) with a reduction of lipid accumulation content (82.8 ± 1.1−132 ± 2% of control) indicated by a reduction of Oil Red O staining (Figure 1A), in which, a significant effect was displayed at a concentration of 1000 µg/mL (Figure 1B). This was significantly correlated to total phenolics content in the MM extract (with a Pearson correlation value 0.908, *p*-value < 0.001) (Figure 3B)

#### 2.3.3. Inhibition of Lipid Accumulation and Reduction of Triglyceride Content

In accordance to the anti-differentiation effect, a bimodal pattern of activity on the intracellular triacylglycerol (triglyceride) contents was exhibited. In groups treated with various concentrations of MM extract, an increase of triglyceride contents (114 to 105 ± 2% of control) was found at low concentrations of MM extract (6.25–250 µg/mL), with significance at concentrations of 6.25 and 125 µg/mL. In contrast, at high concentrations (500 and 1000 µg/mL of MM extract) a significantly reduced intracellular triglyceride content 52.8 ± 1.4 and 29.0 ± 3.3% of control was seen (Figure 1B). This was significantly correlated to anti-differentiation activity (with Pearson correlation value 0.846, *p*-value < 0.001) and total phenolics content in the MM extract (with Pearson correlation value 0.640, *p*-value < 0.005) (Figure 3B).

#### 2.3.4. Adipocyte lipolysis Induction as Increasing of Released Glycerol Content

**Figure 2:**
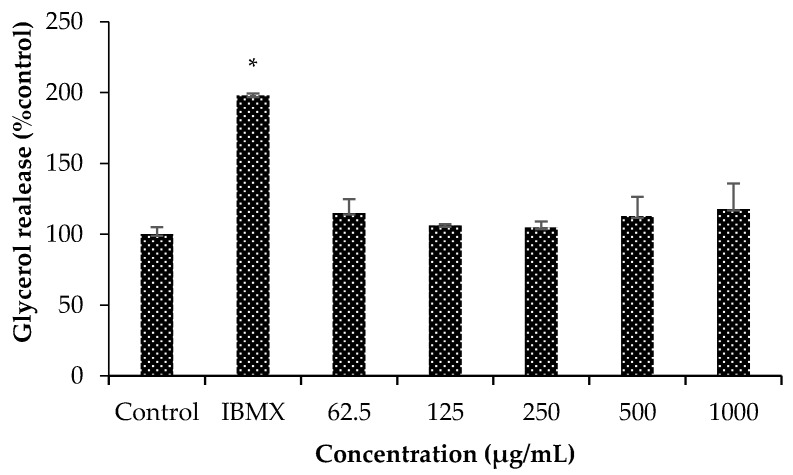
Mild degree of lipolysis induction on 3T3-L1 adipocyte of *Antidesma bunius* L. (MM) extract was found with increasing trend of released glycerol contents. In contrast, 3-isobutyl-1-methylxanthine (IBMX), the reference lipolytic agent, exhibited significant induction on adipocyte lipolysis (*) with *p*-value < 0.05 when compared to control.

Lipolysis induction was indicated by the increase of released glycerol content resulting from degradation of intracellular triglycerides. The increase of lipolysis will lead to a reduction of adipocyte size and number. The result indicated mild activity of MM extract on adipocyte lipolysis. This was demonstrated by a slightly rise of released glycerol contents, ranging between 105 ± 4 to 118 ± 18% of control, in all treated groups, however, these was not significant when compared to the control. In contrast, these obviously differed from IBMX, a lipolytic agent which could significantly induce adipocyte lipolysis with released glycerol contents as high as 198 ± 1 of control (*) with *p*-value < 0.05 when compared to the control (Figure 2).

## 3. Discussion

*Antidesma bunius* L. fruit is an indigenous edible fruit which fresh fruit or processed food products including wine, jam, concentrated juice, and candy have are widely consumed and on-shelf products available in Thailand. Therefore, the scientific data of its health effects and bio-active phytochemical constituents are useful for public perception and marketing value creation. The results from this research work demonstrated the substantial yield of *Antidesma bunius* L. fruit (MM) extract with 12.08% *w*/*w* of fresh weight, composed of phenolics, flavonoids and anthocyanins as its predominant phytochemical components. In addition, anti-oxidant activity in both the DPPH assay and TBAR assay was found with different degrees and susceptibility of action (Table 1). The MM extract exhibited desirable anti-oxidation activity in the DPPH assay, this may imply the contribution of the predominant chemical constituents such as cyanidin-3-glucoside (C3G) and catechin, demonstrated by HPLC-PDA analysis (Table 2) which are known as potent hydrophilic anti-oxidants [12,13]. This result was found in harmony with previous reports, in which catechin and procyanidins were the major components of *Antidesma bunius* L. fruit juice samples amongst others (gallic acid, caffeic acid, rutin, *trans*-resveratrol, quercetin, naringenin, etc.) which exhibited potent hypoglycemic effects in both *in vitro* and *in vivo* models [14,15]. However, there are still some other unidentified constituents in the MM extract that warrant further elucidation of its phytochemical profile and related biological markers. Anti-oxidation is nowadays considered as an empirical beneficial function of medicinal plants and foods for chronic disease prevention and health promotion. Relatively, benefits of antioxidants for obesity therapy involving inhibition of adipogenesis-inducing factors like lipid peroxidation and inflammatory cascades are suggested [16,17,18]. Coincidently, associations between anti-oxidation in a lipophilic study model, reduction of TBARS production, and anti-adipogenic effects in animal study models of *Nelumbo nucifera* [19] and *Plantago albicans* extract [20] have been reported. Recently, Udomkasemsab, et al. [21] revealed the interesting capacity of *Antidesma bunius* fruit extract in improving of glucose metabolism, triglyceride levels, and splenic lesions in high-fat diet-induced hypercholesterolemic rats that involved amelioration of oxidative stress and inflammation. 

The digestive system is another target for anti-obesogenicity via inhibition on two key enzyme inhibitors—Orlistat and Acarbose—widely used as anti-obesity drugs. The inhibitory effect on amylase and lipase enzyme activity of MM extract was manifest, and flavonoid derivatives in the MM extract were assumed to contribute to these activities since they has been reported to possess a wide range of biological activities including regulation of glucose and lipid absorption via inhibition of pancreatic lipase, α-glucosidase and α-amylase enzyme activity [22,23], however, this is in a manner of structure-related potency of action [24,25]. In addition, anti-diabetic activities related to anti-α-glucosidase effect, anti-oxidation, and improving insulin secretion of *Antidesma bunius* L. fruit and seed extract were evidenced by preceding reports [10,26], yet, reports regarding the effect on lipase enzyme of MM extract have been very limited. Therefore, as the earliest evidence, this present study has demonstrated more activity on lipase enzyme, rather than amylase, of MM extract with IC_50_ 90.7 ± 4.1 µg/mL and 14.3 ± 4.1 µg orlistat equiv./mg extract. The attribution to its chemical components—phenolics, proanthocyanins, and anthocyanins—among which cyanidin-3-glucoside showed inhibitory effects on lipase enzyme higher than other glycoside forms, chlorogenic acid had good inhibitory effect to α-amylase enzyme and was superior than cyanidin glycosides [3], a proanthocyanin inhibition of both α-amylase and lipase enzymes in association to polyphenols, including catechin, was denoted [27]. However, the mechanism and inhibitory kinetics regarding lipase and amylase enzyme inhibition of MM extract remains unclear. MM fruit extract is suggested as a promising hypolipidemic and anti-obesity agent involving inhibition on carbohydrate and lipid digestion and absorption via anti-amylase and anti-lipase activity with desirable potency when compared to reference drugs (orlistat with IC_50_ = 1.30 ± 0.17 µg/mL and acarbose with IC_50_ = 13.8 ± 0.4 µg/mL, Table 3).

**Figure 3:**
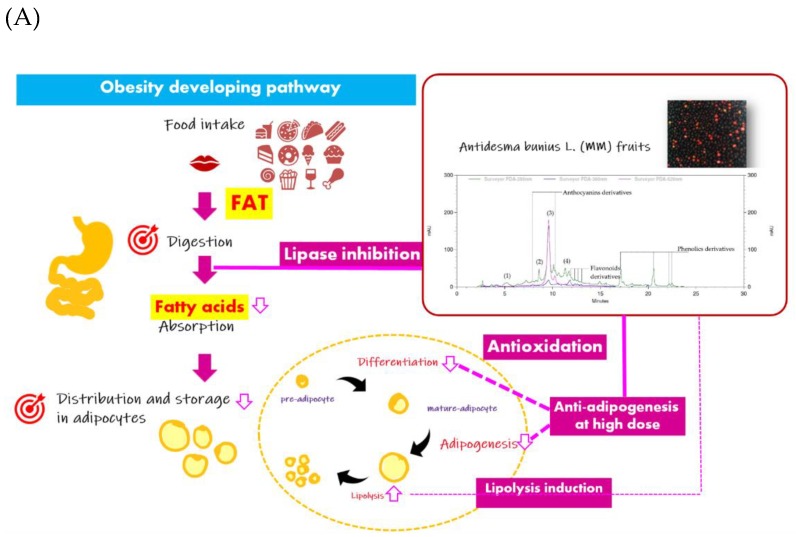

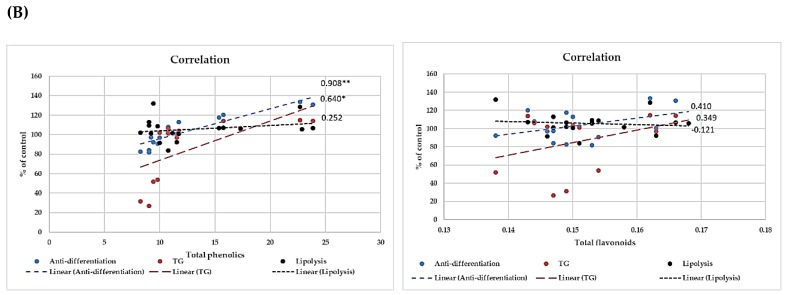
Summary of anti-obesity potential of *Antidesma bunius* L. fruit (MM) extract based on the obtained overall results including (i) the predominant phytochemical components of cyanidin-3-glucoside and catechin and (ii) the key finding bioactivities which promisingly imply the benefits of MM extract for obesity prevention and treatment that would diminish the obesity developing pathway through (iii) inhibition of lipid digestion—anti-lipase enzyme activity; (iv) anti-oxidation in both hydrophilic and lipophilic testing models; (v) interruption of hypertrophic and hyperplasic adipose tissue—anti-differentiation and anti-adipogenesis (anti-lipogenesis) as well as lipolysis induction in adipocytes (**A**), in which, anti-differentiation and anti-adipogenesis (anti-lipogenesis indicated by intracellular triglyceride (TG) content) were significantly correlated to total phenolics contents in MM extract at *p*-value < 0.01 (**) and < 0.05 (*), respectively (**B**).

A bimodal pattern of activity on adipogenesis of MM was revealed, with significant inhibitory effects at high concentrations of MM extract (at 1000 µg/mL), yet paradoxically, a slightly stimulating effect on pre-adipocyte differentiation at low concentrations was detected (Figure 1A,B). This was assumed to be due to particular nutrition and phytochemical components that possess adipogenicity such as sugars and anthocyanins [28,29], however, the actual activity of anthocyanins is still controversial. Sugar, a precursor of triglyceride synthesis in adipocytes prior to a process of lipid storage, is a naturally major nutrient in *Antidesma bunius* L. fruits [30]. In addition, cyanidin-3-glucoside (C3G)—a major species amongst other members in anthocyanins class, and responsible for MM’s purple color [9,15,31], highly found in the studied MM extract—was reported to have adipogenic activity via induction of adipocyte phenotypic changes that result in increasing multi-locular lipid droplets and mitochondrial content in association to up-regulated mitochondrial gene expression [29]. Paradoxically, a high concentration of MM extract (1000 µg/mL) showed significant anti-adipogenesis by reduction of Oil Red O staining and intracellular triglyceride content (Figure 1A,B) and these were strongly correlated with the total phenolics contents (90.8% correlation; Pearson correlation value was 0.908 with *p*-value < 0.01), rather than total flavonoids contents (Figure 3B). This suggested the matter of concentration to effectively achieve anti-adipogenic effect and the important role of phenolics in regulation of intracellular adipogenesis. At this point, to establish the anti-adipogenic factors contained in MM extract, a sufficient concentration of anti-adipogenic active constituents—such as catechin which could suppress adipocyte differentiation by down-regulation of PPAR γ2, C/EBPα, and GLUT4 [32]—may be needed. This discovery implies the versatility of MM extract for health applications related to intake doses, for instance, at a low concentration MM would be beneficial for anti-aging purposes by enhancing and stimulating an increase the cell proliferation. However, further research on identification of complete chemical composition, and the anti-adipogenic and adipogenic properties thereof contained in MM extract as well as their physiological effects and effective doses should be accounted for in order to deliver useful information for further development of products and applications thereof. 

A lipolytic inductive effect of MM extract was detected to a mild degree. Supposedly, this may be result from an inhibitory effect on adipocyte lipolysis of C3G, supported by previous reports in which lipolysis inhibition was demonstrated by reduction of FFAs and glycerol-released contents from adipocytes with a dose- and time-dependent manner under hyperglycemic conditions [33,34]. Also, its mechanism of action via increasing the activity of AMP-activated protein kinase and regulating FoxO1-mediated transcription of adipose triglyceride lipase (ATGL), and thus inhibition of adipocyte lipolysis, has been mentioned [34,35]. Therefore, *Antidesma bunius* L. (MM) was discovered to be a potential anti-obesity agent with health benefits involving multiple targets throughout the obesity development pathway; these include anti-oxidation, lipase inhibition, anti-adipogenesis and mild lipolysis induction (Figure 3A). Relatively, the preventive anti-obesity applications of MM extract with the activity targets related to fat digestion in the gastrointestinal tract and storage in adipose tissue was suggested. Interestingly, a bimodal manner of activity on adipogenensis has been suggested, indicating the necessary of further investigation in *in vivo* and clinical studies to deliver additional useful information for product development and proper applications.

## 4. Materials and Methods

### 4.1. Materials

Porcine pancreatic lipase, porcine pancreatic α-amylase, 4-nitrophenyl butyrate (*p*-NPB), 3,5-dinitrosalicylic acid, acarbose, orlistat, calcium chloride, Folin-Ciocalteu reagent, quercetin, aluminum chloride, ferrous sulfate, sodium carbonate, 3-(*N*-morpholino)propanesulfonic acid (MOPS), 2,2-diphenyl-1-picrylhydrazyl (DPPH), 2-thiobarbituric acid (TBA), butanol, Dulbecco’s modified Eagle’s medium (DMEM), fetal calf serum (FCS), fetal bovine serum (FBS) and penicillin-streptomycin mixture were purchased from Invitrogen (Carlsbad, CA, USA). Dexamethasone (DEX), 3-isobutyl-1-methylxanthine (IBMX), butylated hydroxytoluene (BHT), insulin, Oil Red O solution, triacylglycerol assay kit, free glycerol reagent, 3-(4,5-dimethylthiazol-2-yl)-2,5-diphenyltetrazolium bromide (MTT), dimethyl sulphoxide (DMSO), 2,4,6-tris(2-pyridyl)-s-triazine (TPTZ), ethylene- diamine tetraacetic acid (EDTA), Triton^TM^ X-100, tris (hydroxymethyl) aminomethane, ascorbic acid (vitamin C), potassium chloride (KCl), gallic acid, cyanidin-3-glucoside (C3G), catechin, protocatechuic acid, acetic acid, sodium acetate, egg yolk, sodium lauryl sulfate (SLS), isopropanol, ethanol, methanol, and 0.25% trypsin were purchased from Sigma (St. Louis, MO, USA). All other chemicals used were of analytical grade and purchased from Ajax Finechem (Auckland, New Zealand). Fresh fruits of Mao Luang (*Antidesma bunius* L.; MM) purchased in September 2017 from a farm in Sakon Nakhon province in Northeastern Thailand. Voucher specimens of MM (AB01-2014) was preserved at the Herbarium of Faculty of Pharmaceutical Sciences, Khon Kaen University, Khon Kaen, Thailand. 

### 4.2. Sample Preparation

Mao Luang (MM) ripe fruits were washed with distilled water, dried under ambient conditions for 2 h, blended with 95% ethanol solvent in a ratio of 1:2 (*w*/*v*) and further macerated at ambient temperature (28 ± 2 °C) for 24 h. Then, the filtrate was collected by filtration through a filter membrane (Whatman^®^ No.1) and concentrated using rotary evaporation (Rotavapor^®^ R-3, Büchi, Flawil, Switzerland) and lyophilization (Scanvac CoolSafe^TM^, Lynge, Denmark). The obtained dried extract powder was weighed to calculate % yield (*w*/*w* fresh weight) and kept at −20 °C temperature until use. 

### 4.3. Determination of Total Phytochemical Contents

#### 4.3.1. Total Phenolics Content Using the Folin Ciocalteu Assay

A modification of the method of Singleton and Rossi [36] with gallic acid as a reference compound was applied. Aqueous sample solutions of various concentrations (0–100 µg/mL) were prepared and used to prepare reaction mixtures in a 96-well plate model. The reaction mixtures, containing sample solution and 50% (*v*/*v*) Folin-Ciocalteu reagent (50 µL of each) and 20% (*w*/*v*) sodium carbonate (125 µL) were incubated 40-min at ambient temperature. The optical absorbance was then determined at 700 nm using a spectrophotometer (Sunrise™ microplate reader, Tecan, Männedorf, Switzerland). Using a similar method, a gallic acid calibration curve was plotted using gallic acid contents (*x*-axis) versus the corresponding absorbances (*y*-axis), then, total phenolics content was extrapolated and expressed as mg gallic acid equivalents (mg GA equiv./g extract).

#### 4.3.2. Total Flavonoids Content Using Aluminum-flavonoid Assay

A modification of the method of Chaiittianan, et al. [37] was used. Aqueous sample solutions of various concentrations were used to prepare a reaction mixture comprising 100 µL of sample solution and 100 µL of 2% (*w*/*v*) AlCl_3_ solution to give a final sample concentration of 0–100 µg/mL. Samples were incubated at room temperature for 30-min, then, the optical absorbance was determined at 437 nm using the spectrophotometer. Using a similar method, a quercetin calibration curve was plotted between quercetin contents (*x*-axis) versus the corresponding absorbances (*y*-axis). Total flavonoids content was extrapolated and expressed as mg quercetin equivalents (mg QE equiv./g extract).

#### 4.3.3. Total Anthocyanins Content Using pH-differential Assay

A modified pH differential method of Lee et al. [38] was used, in which aqueous sample solutions of MM at a concentration of 20 µg/mL in KCl (pH 1) and sodium acetate buffer solution (pH 4) were independently prepared. Then, the optical absorbance of the sample solutions was measured at 510 and 700 nm with nine replications for each measurement using the spectrophotometer. The obtained absorbances were used to calculate the total anthocyanins content using the equation below and expressed as mg cyanidin-3-glucoside equivalents (mg C3G equiv./g extract).

Anthocyanin content (mg/L) = (A × MW × DF × 10^3^)/(ε × L) (1)

where A = differential absorbance at two different factors such as wavelength and pH:

(A_510_ nm−A_700_ nm)_pH 1.0_−(A_510_ nm−A_700_ nm)_pH 4.5_ (2)

where MW = molecular weight of cyanidin-3-glucoside 449.2 g/mol, ε = molarity absorptivity of 26,900 L/(mol·cm), L = light path width of 1 cm, DF = dilution factor and 10^3^ = factor for conversion from g to mg.

### 4.4. Determination of Anti-oxidation Activity Using DPPH and TBARS Assays

A DPPH assay based on a modification of the method of Chu et al. [39] was applied in a 96-well plate model, whereby various concentrations of methanolic sample solutions were prepared and used to constitute reaction mixtures comprised of sample solution (150 µL) and 0.1 mM DPPH solution (50 µL). Samples were incubated for 15-min under ambient conditions. Optical absorbance at 570 nm was measured using the spectrophotometer. The anti-oxidative effect of each sample was expressed as mg vitamin C equivalents (mg Vit C equiv./g extract) as well as 50% inhibitory concentration (IC_50_). A TBARS assay based on a modification of the method of Chaiittianan, et al. [37] was conducted in a 96-well plate model to determine the inhibitory effect on thiobarbituric acid reactive substance (TBARS) production of each sample, compared to butylated hydroxytoluene (BHT), a reference anti-lipid peroxidaton compound. Methanolic sample solutions were prepared at various concentrations and used to make reaction mixtures comprising sample solution (90 µL), 30% (*w*/*v*) egg yolk in 1.5% (*v*/*v*) KCl aqueous solution (90 µL) and 1 mM FeSO_4_ (60 µL), followed by 1-h incubation at 37 °C temperature. Then, 300 µL of 20% (*v*/*v*) acetic acid and 300 µL of 0.8% (*w*/*v*) thiobarbituric acid in SLS 1.1% (*w*/*v*) were added to the reaction mixtures, followed by 10-min incubation at 90 °C temperature before adding 500 µL of butanol. The supernatant was collected and the optical absorbance measured at 532 nm using the spectrophotometer. The anti-oxidation activity of each sample was calculated and expressed in mg BHT equivalents (mg BHT equiv./g extract) and IC_50_.

### 4.5. HPLC-UV Analysis of the Phytochemical Contents

HPLC analysis was conducted compared to standard compounds: phenolics (gallic acid, catechin, and protocatechuic acid) and anthocyanins (cyanidin-3-glucoside), Methanolic solutions of standard compound mixture (various concentrations from 1.56–50 μg/mL) and MM extract (concentration of 10 mg/mL) were independently prepared and used for qualitative and quantitative analysis on a LC-2030C PDA HPLC system (Shimadzu, Kyoto, Japan), equipped with a C18 Hypersil Gold PFP column (particle size 5 μm, dimension 4 × 250 mm) and diode-array UV detection at wavelengths of 280, 360 and 520 nm. For each analysis a gradient mobile phase system was used at a flow rate of 1 mL/min, comprised of solution A (0.1% *v*/*v* formic acid in deionized water) and solution B (methanol) with a gradient of increasing ratio of solution B to 100% within 15 min (0.01–15 min), hold at 100% solution B for 10 min (15.01–25 min), and decreasing to 0% solution B within 5 min (25.01–30 min), respectively. Ten µL injections of sample solutions were done. Prior to sample analysis, the calibration curves of standard compounds, based on plots of average peak area (*y*-axis) vs. the corresponding concentration (*x*-axis) to get linear relationships expressed by a linear equation (y = ax + b) and regression (r^2^)-using standard compound mixture solutions were simultaneously established and used for quantitative analysis. Then, each sample was analyzed in independent triplicates. As the result, the obtained chromatograms with details of peak retention time (min) and peak area of each compound were subsequently used for peak identification and content calculation, compared to standard compounds. Each chemical constituent content was extrapolated from the developed calibration curves of standard compounds and expressed in term of mg per gram extract (mg/g extract).

### 4.6. Determination of Inhibitory Activity on Lipase and Amylase Enzyme

#### 4.6.1. Effect on Lipase Enzyme

A modified method of Lee, et al. [40] was applied. Various concentrations of sample (0–1000 µg/mL) or orlistat, a reference anti-lipase drug, (0–10 µg/mL) solution were prepared in Tris buffer solution (100 mM Tris-HCl and 5 mM CaCl_2_, pH 7.0) The reaction mixture was constituted which comprised of 80 µL of sample (or orlistat) solution, 80 µL of 5 mg/mL (26 units) of lipase enzyme solution (dissolved in 10 mM morpholinepropanesulfonic acid (MOPS) and 1 mM EDTA pH 6.8 and Tris buffer solution) and 80 µL of Tris buffer solution, and 100 µL of 2.5 mM *p*-nitrophenyl butyrate (*p-*NPB) (dissolved in dimethylformamide) and followed by 30-min incubation at 37 °C. The optical absorbance was detected at 403 nm using the spectrophotometer. Inhibitory activity was expressed as calculated IC_50_.

#### 4.6.2. Effect on α-Amylase Enzyme

A modified method of Worsztynowicz, et al. [3] was used. Various concentrations of sample (0–1000 µg/mL), acarbose—a reference anti-amylase drug (0–200 µg/mL)—α-amylase enzyme solution (1.3 mg/mL) and 1% corn starch solution were prepared in 0.02 M phosphate buffer solution (pH 6.9). Then, the reaction mixture was constituted by mixing 60 µL of sample (or acarbose) solution, 60 µL (1.325 units) of amylase enzyme solution and 60 µL of 1% corn starch solution and followed by 15-min incubation at 37 °C. To detect the result, 80 µL of DNS solution was added into the reaction mixture and incubated at 80 °C for 10 min. Then, the optical absorbance was measured at 540 nm using the spectrophotometer. Inhibitory activity was calculated and expressed as calculated IC_50_.

### 4.7. Determination of Anti-obesity Potential via Interruption of Adipocyte Cell Cycle

#### 4.7.1. Cell Viability Using MTT Assay

3T3-L1 preadipocytes were seeded into the wells of 24-well plates at a density of 40,000 cells/well and cultured at 37 °C temperature in a humidified atmosphere of 5% CO_2_ until 80% confluence. Then they were treated with various concentrations of sample extract solution which was dissolved in PM culture medium and incubated at 37 °C in humidified atmosphere of 5% CO_2_ for 48 h. MTT assay cell viability was determined by addition of 200 µL of MTT solution (5 mg/mL in phosphate-buffer saline) to each well and incubation at 37 °C for 4 h. After removal of the culture medium, 500 µL of DMSO was added to each well, followed by 15-min incubation at room temperature. Then, the optical absorbance was determined at 560 nm using the spectrophotometer. Compared to the control (untreated group), the results were expressed as % cell viability, the eligible concentrations for further experiment were accounted when % cell viability >80.

#### 4.7.2. Anti-adipogenesis in 3T3-L1 Adipocytes

##### Culture Conditions for Pre-adipocyte Maintenance and Differentiation

Followed a modification of the method of Chaiittianan et al. [37], 3T3-L1 murine pre-adipocytes (ATCC CL173), the 4th sub-passaged cell culture, maintained in pre-adipocyte medium (PM), 10% fetal calf serum (FCS) supplemented Dulbecco’s modified eagle medium (DMEM) containing 1% penicillin–streptomycin, at 37 °C temperature in humidified atmosphere of 5% CO_2_ until reached 90% confluence, were used in the experiments. Cells were seeded into 24-well plates at a density of 40,000 cells/well and cultured and maintained under the same condition until 80% confluence. Subsequently, differentiate induction was conducted by sequential treatment of specific media including differentiation medium I (DMEM, 10% FCS, 1 µM dexamethasone (DEX), 500 µM 3-isobutyl-1-methylxanthine (IBMX), and 10 µg/mL insulin) and differentiation medium II (DMEM, 10% FCS and 10 µg/mL insulin) and medium III (DMEM, 10% fetal bovine serum; FBS) in the manner of presenting of the MM extract (concentration range of 0–1000 µg/mL) and followed by 48-h incubation in each medium for determination of anti-adipogenesis activity, differently, of no MM extract in determination of lipolysis induction. Compared to the control, the adipogenesis was determined based on the degree of cell differentiation and lipid accumulation detected by Oil Red O staining and triacylglycerol determination kit. Similarly, the differentiated adipocytes were used which in the preparation was slightly different when differentiated. 

##### Oil Red O Staining

To visualize cellular differentiation and lipid accumulation, the differentiated adipocytes were fixed with 10% (*v*/*v*) formalin for 30 min, rinsed with PBS (pH 7.4), stained with freshly prepared 0.5% (*w*/*v*) Oil Red O solution at 37 °C for 1 h, and the stained cells were photographed using an inverted microscope (Axio Vert.A1 FLLED, ZEISS^®^, Jena, Germany) to determine the accumulated lipid content, the retained dye in adipocytes was extracted with 100% isopropanol and quantified by measuring the optical absorbance at 510 nm using the spectrophotometer. The inhibitory effect on cell differentiation of each sample was presented by relative lipid accumulation content compared to the control (% of control) 

##### Triglyceride (Triacylglycerol) Determination

Differentiated adipocytes were washed twice with ice-cold PBS (pH 7.4), scraped in 300 µL of lysis buffer (0.15 M NaCl, 10 mM EDTA, 0.1% Triton^TM^ X-100, 50 mM Tris buffer; pH 7.4) and followed by 10-min sonication (ultrasonicator, Witeg Labortechnik GmbH, Wertheim, Germany). The lysed cell mixture was centrifuged at 4 °C, 12,000× *g* for 10 min to collect the supernatant for quantitative analysis of the triglyceride (triacylglycerol) content by using serum triacylglycerol determination kit. The optical absorbance at 540 nm were examined using the spectrophotometer. The inhibition on lipid accumulation was presented by relative triacylglycerol content of each sample compared to the control (% of control).

#### 4.7.3. Lipolysis Induction by Glycerol Released Assay

The differentiated adipocytes were incubated overnight in phenol red-free DMEM supplemented with 0.5% (*v*/*v*) FBS at 37 °C in humidified atmosphere of 5% CO_2_. Then, the cells were treated with phenol red-free DMEM (as control), 50 µM IBMX (as a reference lipolytic agent) and sample solution at designated concentrations and incubated for another 24 h. To investigate the effect in lipolysis, the supernatant of each was collected, heated at 70 °C for 10 min, mixed with free glycerol reagent in the ratio of 1:1 (*v*/*v*) and followed by 15-min incubation. Then, the optical absorbance was determined at 540 nm using the spectrophotometer). The lipolytic induction of each sample was presented by relative glycerol released content when compared to the control (% of control).

### 4.8. Statistical Analysis

Results were expressed as mean ± standard deviation (SD) of values that obtained from at least triplication of independent studies. Statistical analysis was done by using one-way ANOVA followed by LSD multiple comparisons for parametric and Mann-Whitney U test for non-parametric data. In addition, the pearson correlation analysis was conducted in SPSS software version 19 (Chicago, IL, USA). The significance was considered at *p*-value less than 0.05 (*p*-value < 0.05).

## 5. Conclusions

*Antidesma bunius* L. (Mao Luang in Thai), a Thai local fruit, which is predominantly composed of phenolics and anthocyanins was promisingly manifested as a natural source of anti-obesity agents accompanied with potential health-promoting benefits. Its properties and potential were evidenced involving multiple targets of action, demonstrated by a desirable degree of antioxidation, anti-lipase enzyme, and anti-adipogenicity at high concentrations. In addition, a remarkable concentration-dependent bimodal manner of action on adipocyte adipogenesis was observed. From the overall results, the health benefits and anti-obesity potential, in particular for preventive manner applications, of *A. bunius* L. have been discovered, suggesting the need for further investigation in physiological systems to define the proper dosage for anti-obesity applications.

## Figures and Tables

**Table 1:** Total phenolics content expressed as mg gallic acid (GA) equiv./g extract, flavonoids expressed as mg quercetin (QE) equiv./g extract, anthocyanins contents expressed as mg cyanidin-3-glucoside (C3G) equiv./g and anti-oxidation expressed as 50% inhibitory concentration (IC_50_) and mg equivalents of vitamin C (Vit C) or butylated hydroxytoluene (BHT)/g extract of *Antidesma bunius* L.

Phytochemical Contents		Equivalent Weight to Reference Compound (mg eq/g Extract)	
**Total phenolics content**		11.57 ± 1.13 mg GA eq/g extract	
Total flavonoids content		0.30 ± 0.00 mg QE eq/g extract	
Total anthocyanins content		3.76 ± 0.19 mg C3G eq/g extract	
Antioxidation activities	IC_50_ (µg/mL)		Equivalent weight to reference compound (mg eq/g extract)
DPPH assay (Vit C, IC_50_ 14.47 ± 0.42 µg/mL)	652.30 ± 5.56		21.81 ± 3.50 mg Vit C eq/g extract
TBAR assay (BHT, IC_50_ 2.16 ± 0.18 µg/mL)	>1000		3.55 ± 0.42 mg BHT eq/g extract

**Table 2:** The chemical composition of *Antidesma bunius* L. (MM) extract was primitively identified based on the principle of chromatographic and spectroscopic identity of each compound compared to that of standard compounds including specific retention time (RT) and UV spectrum with specific maximal wavelength (λmax) under the HPLC-PDA system. There were more than 20 peaks in MM HPLC chromatogram within 30-min run. Among them, four predominant peaks of gallic acid, catechin, cyanidin-3-glucoside and protocatechuic acid (peak# 1–4) were successfully identified and subsequently quantified by extrapolation from the calibration curves (y = ax + b, linear regression coefficient (r^2^)) and expressed as mg/g extract.

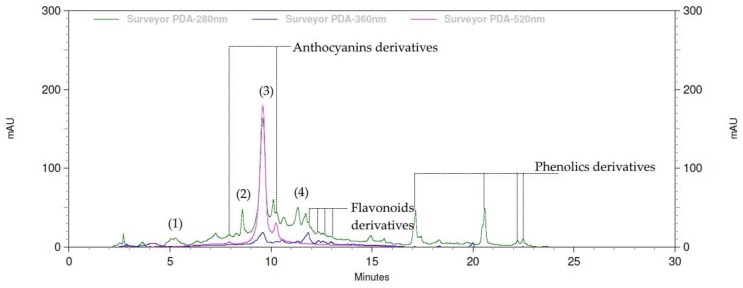					
Peak#	Identification	RT (min)	λ_max_ (nm)	Linear Equation y = ax + b (r^2^)	Content (mg/g Extract)
1	Gallic acid	5.167	203/224**/271*	y = 39115x − 26163 (0.999)	0.26 ± 0.01
2	Catechin	8.250	206/234*/279**	y = 6513x − 5080 (0.999)	2.26 ± 0.61
3	Cynidin-3-glucoside	9.600	244/276**/529*	y = 17572x (0.995)	13.38 ± 0.72
4	Protocatechuic acid	11.833	202/261*/295**	y = 73467x − 40696 (0.999)	0.24 ± 0.01

* primary band, ** secondary band of the UV spectrum.

**Table 3:** Inhibitory effect on α-amylase and lipase enzyme of *Antidesma bunius* L. extract expressed as 50% inhibitory concentration (IC_50_) or maximal inhibitory effect (%inhibition) at highest concentration (1000 µg/mL) and equivalent weight to acarbose (or orlistat) reference drug.

Parameters	Lipase Enzyme	Amylase Enzyme
Reference drug	Orlistat (IC_50_ = 1.30 ± 0.17 µg/mL)	Acarbose (IC_50_ = 13.83 ± 0.38 µg/mL)
IC_50_	90.75 ± 4.12 µg/mL ^†^	n.d. (Maximal inhibition 44.25 ± 0.86%^§^)
Reference drug equivalent weight	14.33 ± 4.12 µg Orlistat eq/mg extract	0.45 ± 0.05 µg Acarbose eq/mg extract

n.d. means not detectable; † IC50 values were calculated from logarithmic equation y = 21.732ln(x) + 46.67(r^2^ = 0.9827) for anti-lipase activity; § IC50 values were calculated from the logarithmic equation y = 11.447ln(x) + 19.83 (r^2^ = 0.9711) for anti-amylase activity.
